# EPR spectroscopy of chlorpromazine-induced free radical formation in normal human melanocytes

**DOI:** 10.1007/s00249-015-1029-6

**Published:** 2015-05-16

**Authors:** Michał Otręba, Magdalena Zdybel, Barbara Pilawa, Artur Beberok, Dorota Wrześniok, Jakub Rok, Ewa Buszman

**Affiliations:** Chair and Department of Pharmaceutical Chemistry, School of Pharmacy with the Division of Laboratory Medicine in Sosnowiec, Medical University of Silesia, Jagiellońska 4, 41-200 Sosnowiec, Poland; Chair and Department of Biophysics, School of Pharmacy with the Division of Laboratory Medicine in Sosnowiec, Medical University of Silesia, Jedności 8, 41-200 Sosnowiec, Poland

**Keywords:** Melanin, Melanocytes, Chlorpromazine, Free radicals, EPR spectroscopy

## Abstract

The purpose of this study was to estimate the effect of chlorpromazine on free radical concentration in HEMn-DP melanocytes using electron paramagnetic resonance (EPR) spectroscopy. It was found that chlorpromazine at concentrations of 1 × 10^−7^ and 1 × 10^−6^ M contributed to the formation of free radicals (*g* values ~2) in a dose-dependent manner. The increase in free radical formation was accompanied by an increase in cytotoxicity, as shown by a tetrazolium assay. Homogeneous broadening of EPR lines, slow spin–lattice relaxation processes, and strong dipolar interactions characterized all the tested cellular samples. The performed examination of free radical formation in cells exposed to different chlorpromazine concentrations confirmed the usefulness of electron paramagnetic resonance spectroscopy to determine the effect of a drug on free radical production in a cellular model system in vitro.

## Introduction

Chlorpromazine, a member of the largest class of first-generation antipsychotic agents—the phenothiazines, is known to cause severe side effects during long-term and/or high-dose therapy. Chlorpromazine is widely used in the treatment of schizophrenia, psychotic disorders, and the manic phase of bipolar disorders, but also demonstrates anticancer, antibacterial, antiviral, antiprionic, and multidrug resistance reversal activity (Motohashi et al. 2000, 2006). Chlorpromazine therapy is associated with extrapyramidal effects, hepatotoxicity, agranulocytosis, hyperprolactinemia, as well as with other side effects such as skin (e.g., jaundice and pigmentation, lichenoid, photosensitivity, pigmentation, subacute lupus erythematosus, toxic epidermal necrolysis, urticaria) and ocular (e.g., retinopathy, cataract) disorders (Anthérieu et al. 2013; Drucker and Rosen 2011; Mitkov et al. 2014; Shahzad et al. 2002; Subashini and Rao 2004; Toler 2004). Oxidative stress plays an important role in the pathogenesis of some diseases such as cholestasis, tardive dyskinesia, cataract, age-related macular degeneration (AMD), and retinopathy (Anthérieu et al. 2013; Toler 2004; Athanasiou et al. 2013; Fletcher 2010; Pillai et al. 2006). Chlorpromazine has been reported to cause pigmentation changes and photosensitivity. The skin changes start as a brown discoloration in the sun-exposed areas (the neck, face, lower legs, and dorsum of the hands) and they progress to a darker gray-blue color (Mitkov et al. 2014). The skin and ocular disorders suggest a potential role of endogenous melanin in the induction of these side effects in pigmented tissues. Our previous studies have shown that chlorpromazine forms stable complexes with melanin (Buszman et al. 2008), but the relation between the drug affinity to melanin and the skin or eye toxicity is not clear.

Melanocytes are highly specialized, dendritic pigment cells, which exist in the skin (epidermis, hair follicles), hair, eye (uveal tract, retinal pigment epithelium), inner ear, brain, lung, heart, and adipose tissue, where they play very important physiological roles (Plonka et al. 2009; Tolleson 2005). Melanins are biopolymers synthesized in melanocytes, in unique lysosome-related organelles—melanosomes, through a physiological process called melanogenesis (Tolleson 2005). Melanins are widely distributed in organism and are responsible for the color of eyes, skin, and hair, as well as the interaction with drugs, influencing therapeutic and toxic effects (Plonka et al. 2009; Tolleson 2005; Otręba et al. 2012). It has been demonstrated that melanins possess superoxide dismutase activity and are able to remove free radicals and reactive oxygen species (ROS) generated in response to UV radiation, chemical substances, and environmental stress. Thus, melanins may protect pigmented tissues against different impairments (protein modifications, DNA damage) induced by oxidative stress (Otręba et al. 2012; Frey et al. 2007; Hoogduijn et al. 2004).

Melanins are paramagnetic polymers with high free radical content (Pasenkiewicz-Gierula and Sealy 1986; Ito and Wakamatsu 2008; Zdybel et al. 2011, 2012; Chodurek et al. 2012, 2013). Spectroscopic studies have shown that free radicals existing in melanins play an important role during binding of drugs to this biopolymer (Zdybel et al. 2013; Beberok et al. 2010; Pilawa et al. 2002; Buszman et al. 2005; Najder-Kozdrowska et al. 2010). The changes of free radical concentration after complexation of melanin by drugs were observed. Moreover, our earlier studies have indicated that various concentrations of fluoroquinolone antibiotics, e.g., ciprofloxacin, lomefloxacin (Beberok et al. 2010), and moxifloxacin (Beberok et al. 2014) modified the free radical concentration in melanin. Free radical formation in melanocytes under chlorpromazine treatment has not been studied by electron paramagnetic spectroscopy so far.

The aim of this study was to determine the effect of chlorpromazine on free radical formation in normal human melanocytes (HEMn-DP), which is very important for the assessment of toxic interactions produced by this drug in living organisms. The changes in free radical concentrations in melanocytes treated with different amounts of chlorpromazine were tested and magnetic interactions in the samples were characterized.

## Materials and methods

### Materials

Chlorpromazine hydrochloride, phosphated-buffered saline (PBS), l-3,4-dihydroxyphenylalanine (l-DOPA), and amphotericin B were purchased from Sigma-Aldrich Inc. (USA). Neomycin sulfate was obtained from Amara (Poland). Penicillin was acquired from Polfa Tarchomin (Poland). Growth medium M-254 and human melanocyte growth supplement-2 (HMGS-2) were obtained from Cascade Biologics (UK). Trypsin/EDTA was obtained from Cytogen (Poland). Cell Proliferation Reagent WST-1 was purchased from Roche GmbH (Germany). The remaining chemicals were produced by POCH S.A. (Poland).

### Preparation of DOPA-melanin

Model DOPA-melanin was obtained by oxidative polymerization of l-DOPA solution (1 mg/ml) in 0.067 mol/l phosphate buffer (pH 8.0) for 48 h, according to the method described earlier (Beberok et al. 2014).

### Cell culture

The normal human epidermal melanocytes, neonatal, dark pigmented (HEMn-DP, Cascade Biologics) were grown according to the manufacturer’s instructions. The cells were cultured in M-254 basal medium supplemented with HMGS-2, penicillin (100 U/ml), neomycin (10 μg/ml), and amphotericin B (0.25 μg/ml) at 37 °C in 5 % CO_2_. All experiments were performed using cells in the passages 6–9.

### Cell viability assay

The viability of melanocytes was evaluated by the WST-1 (4-[3-(4-iodophenyl)-2-(4-nitrophenyl)-2H-5-tetrazolio]-1,3-benzene disulfonate) colorimetric assay. WST-1 is a water-soluble tetrazolium salt, the rate of WST-1 cleavage by mitochondrial dehydrogenases correlates with the number of viable cells. In brief, 5000 cells per well were placed in a 96-well microplate in a supplemented M-254 growth medium and incubated at 37 °C and 5 % CO_2_ for 48 h. Then the medium was removed and the cells were treated with chlorpromazine solutions in a concentration range from 1 × 10^−8^ to 1 × 10^−5^ M. After 21-h incubation, 10 μl of WST-1 was added to 100 μl of culture medium in each well, and the incubation was continued for 3 h. The absorbance of the samples was measured at 440 nm with a reference wavelength of 650 nm, against the controls (the same cells but not treated with chlorpromazine) using a microplate reader UVM 340 (Biogenet, Poland). The controls were normalized to 100 % for each assay and treatments were expressed as the percentage of the controls.

### EPR measurements

Free radicals in HEMn-DP control cells and the cells treated with chlorpromazine were examined at room temperature by the use of an X-band (9.3 GHz) electron paramagnetic resonance (EPR) spectroscopy. The EPR spectra were detected for the cell samples located in thin-walled glass tubes with an external diameter of 1 mm. The volume of the cells in these tubes was determined. The empty glass tubes were free of paramagnetic impurities and they did not reveal EPR signals. The measurements were performed by the Radiopan (Poznań, Poland) EPR spectrometer and the Rapid Scan Unit of Jagmar (Kraków, Poland). Magnetic modulation was 100 kHz. The total microwave power (*M*_0_) produced by the klystron of the spectrometer was 70 mW. The influence of microwave power, in the range of 2.2–70 mW, on EPR spectra was determined. Spectroscopic programs SWAMP by Jagmar (Kraków, Poland) and LabVIEW 8.5 by National Instruments were used during the measurements and to the analysis of EPR spectra.

For EPR spectra, the following parameters: g factors, amplitudes (*A*), integral intensities (*I*) and linewidths (Δ*B*_pp_), were determined. *g* values were calculated from the electron paramagnetic resonance condition according to the formula (Wertz and Bolton 1986):

$$ g = h\nu /\mu_{\text{B}} B_{\text{r}} $$

where *h* is the Planck constant; *ν* microwave frequency; *μ*_B_ Bohr magneton; and *B*_r-_induction of resonance magnetic field. Microwave frequency (*ν*) was directly measured by MCM101 recorder of EPRAD Firm (Poznań, Poland).

Integral intensity (*I*) is defined as the area under the absorption curve (Wertz and Bolton 1986). The values of integral intensities were calculated by double integration of the first-derivative EPR spectra. Free radical concentrations in the cell samples were proportional to the integral intensities (*I*). The integral intensities (*I*) for the tested samples were normalized by dividing their values by the volume of the samples and additionally normalized to protein content (*I*/mg protein).

The influence of microwave power (*M*) on the amplitudes (*A*) and linewidths (Δ*B*_pp_) of the EPR spectra of HEMn-DP cells (control and treated with chlorpromazine) was examined. The correlations between amplitudes (*A*), linewidths (Δ*B*_pp_), and microwave power (*M*) give information about homogeneous or inhomogeneous line broadening. For homogeneous broadening of spectral lines, the amplitude (*A*) increases with increasing microwave power (*M*), reaches maximum, and after its value decreases for higher microwave powers (Wertz and Bolton 1986; Eaton et al. 1998). Linewidth (Δ*B*_pp_) of the homogeneously broadened EPR lines increases with rising microwave power (*M*) (Wertz and Bolton 1986; Eaton et al. 1998). For inhomogeneous broadened EPR lines, amplitude (*A*) does not change with microwave power after reaching its maximal value and linewidth (Δ*B*_pp_) does not depend on microwave power (Wertz and Bolton 1986; Eaton et al. 1998). The changes of amplitudes with increasing microwave power characterize the spin–lattice relaxation processes in the samples. The power of EPR line microwave saturation increases with faster spin–lattice relaxation processes (Wertz and Bolton 1986; Eaton et al. 1998).

## Results and discussion

One of the reasons for the occurrence of undesirable side effects after phenothiazine derivatives treatment may be the ability of these drugs to induce oxidative stress in cells. The formation of drug–melanin complexes may lead to the accumulation of drugs in pigmented tissues and a reduction of melanin capacity to free radical trapping (Shahzad et al. 2002; Richa and Yazbek 2010). This, in turn, may affect the melanin biosynthesis as well as the activity of antioxidant enzymes in melanocytes, which may induce cell apoptosis and toxic effects. The human epidermis is a special target for oxidative stress owing to its constant exposure to high levels of ROS produced by physical, chemical, and biological reactions (Gibbons et al. 2006). The superoxide radical anion, lipid peroxides, hydroxyl radicals, and hydrogen peroxide are produced in oxidative processes of cellular metabolism. They are accumulated in tissues upon aging as well as under pathological conditions leading to oxidative stress (Mattila et al. 2007). Oxidative stress is generated by an imbalance between the production of ROS and activity of endogenous free radical scavengers (Frey et al. 2007). ROS are known to damage various cellular components, including membrane lipids, proteins, DNA, and thereby contribute to cellular dysfunction (Pillai et al. 2006; Frey et al. 2007; Hoogduijn et al. 2004). Overproduction of ROS has been linked with several major diseases, such as cancer, diabetes, schizophrenia, depigmentation (e.g., hair graying and vitiligo) and neurodegenerative disorders (mostly both Parkinson’s and Alzheimer’s diseases) (Mattila et al. 2007; Kim and Lee 2013). It has also been shown that chronic treatment with typical antipsychotics increases free radical production and oxidative stress by altering the levels of antioxidant enzymes and causing oxidative injury (Pillai et al. 2006; Naidu et al. 2002).

We previously demonstrated that chlorpromazine forms stable complexes with ocular as well as synthetic melanin characterized by two classes of independent binding sites: strong binding sites (*n*_1_) with the association constant *K*_1_ ~10^4^ M^−1^ and weak binding sites (*n*_2_) with *K*_2_ ~10^3^ M^−1^ (Buszman et al. 2008). The total number of binding sites (*n*_1_ + *n*_2_) was about 0.6 µmol drug/mg melanin. The relation between the affinity of phenothiazine derivatives to melanin and their toxicity has been suggested, but is not well documented. However, the ability of chlorpromazine to form complexes with melanin in vitro may be one of the reasons for its in vivo toxicity on pigmented cells, as a result of its interaction with melanin.

Cell viability was determined by the WST-1 test after 24-h incubation with chlorpromazine in a concentration range from 1 × 10^−8^ to 1 × 10^−5^ M. It has been demonstrated that chlorpromazine induces concentration-dependent loss in cell viability (Table 1). Treatment of cells with 1 × 10^−7^, 1 × 10^−6^, 2.5 × 10^−6^, 5 × 10^−6^, 7.5 × 10^−6^, and 1 × 10^−5^ M of chlorpromazine for 24 h has led to the loss of 8.3, 25.3, 49.6, 68.7, 81.0, and 95.0 % in the melanocytes viability, respectively. The value of EC_50_ (the concentration of a drug that produces loss in cell viability by 50 %) was calculated to be 2.53 × 10^−6^ M. At the lowest studied drug concentration (1 × 10^−8^ M), the loss in cell viability was not observed.

**Table 1:** The effect of chlorpromazine on viability of melanocytes

Chlorpromazine concentration (M)	Cell viability (%) ± SEM
1.0 × 10^−8^	100.6 ± 3.2
1.0 × 10^−7^	91.7 ± 0.8
1.0 × 10^−6^	74.7 ± 0.9
2.5 × 10^−6^	50.4 ± 3.9
5.0 × 10^−6^	31.3 ± 3.7
7.5 × 10^−6^	19.0 ± 2.5
1.0 × 10^−5^	5.0 ± 2.4

Cells were treated with various chlorpromazine concentrations (ranging from 1 × 10^−8^ to 1 × 10^−5^ M) and examined by WST-1 assay. Data are expressed as percentage of cell viability. Mean values ± SEM from three independent experiments (*n* = 3) performed in triplicate are presented

**Fig. 1:**
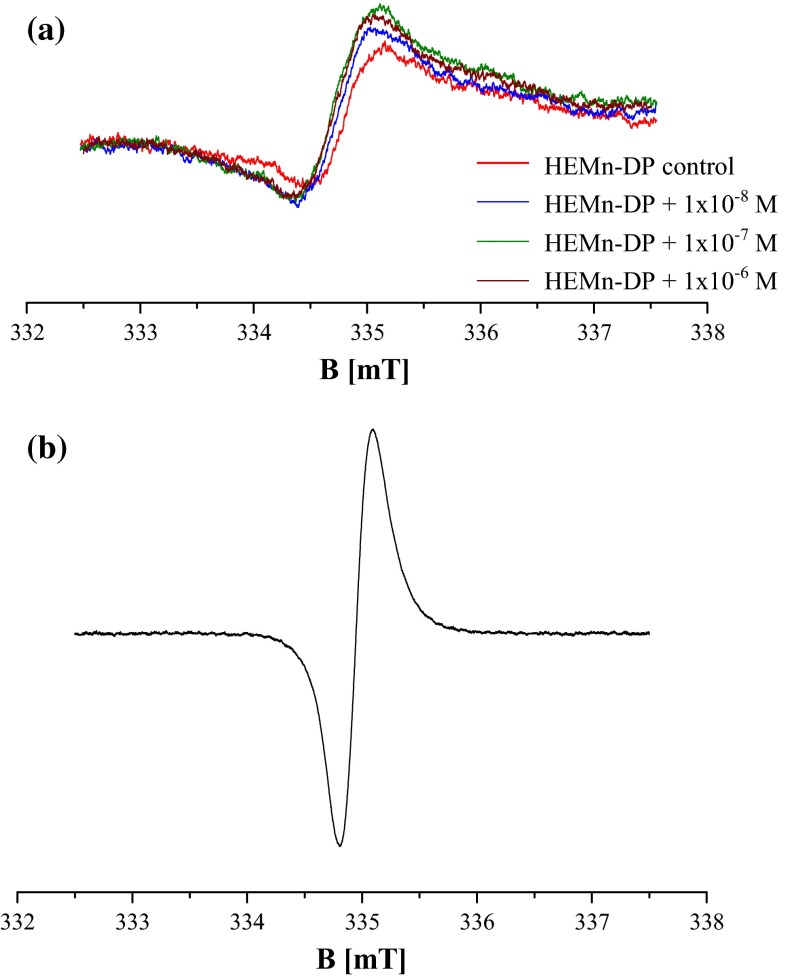
EPR spectra of HEMn-DP control cells and the cells treated with chlorpromazine in concentrations of 1 × 10^−8^, 1 × 10^−7^, and 1 × 10^−6^ M (**a**) and DOPA-melanin (**b**). The EPR spectra were measured with microwave power of 2.2 mW. *B* magnetic induction

The ability of chlorpromazine to induce free radical formation in normal human melanocytes was estimated by the use of EPR spectroscopy. Electron paramagnetic resonance examination pointed out that free radicals exist in all the tested cell samples. EPR lines were detected for both the HEMn-DP control cells and HEMn-DP cells treated with different concentrations of chlorpromazine (Fig. 1a). The EPR spectra revealed a similar shape to the EPR spectrum of model eumelanin—DOPA-melanin, which is presented in Fig. 1b. EPR spectra of all the tested samples (Fig. 1a) as well as DOPA-melanin (Fig. 1b) were single broad asymmetric lines. It indicates that melanin free radicals are mainly responsible for EPR signals of the examined melanocytes. The EPR spectra of control cells and cells treated with the drug were broad lines with the parameters dependent on the used chlorpromazine concentration in the cell culture. The parameters of the EPR spectra of control HEMn-DP cells and the cells cultured with chlorpromazine with concentrations 1 × 10^−8^, 1 × 10^−7^, and 1 × 10^−6^ M are compared in Table 2. Table 2 contains integral intensities (*I*), integral intensities normalized to protein content (*I*/mg protein), and linewidths (Δ*B*_pp_) for the first-derivative lines of the tested cells.

**Table 2:** Integral intensities (*I*), integral intensities normalized to protein content (*I*/mg protein), and linewidths (Δ*B*_pp_) of EPR spectra of the studied human epidermal melanocytes HEMn-DP. Free radical concentrations in the cells are proportional to the integral intensities (*I*)

Sample	*I* (a. u.) ± 0.2	*I*/mg protein (×10^2^)	Δ*B*_pp_ (mT) ± 0.02
HEMn-DP control	3.7	1.6	0.63
HEMn-DP + 1 × 10^−8^ M	3.8	1.7	0.67
HEMn-DP + 1 × 10^−7^ M	5.0	2.2	0.73
HEMn-DP + 1 × 10^−6^ M	4.5	2.1	0.71

The data for the control cells and cells treated with chlorpromazine are shown. The EPR spectra were measured with microwave power of 2.2 mW

For the analyzed samples, *g* values were near 2, which is the value characteristic for free radicals (Wertz and Bolton 1986; Eaton et al. 1998). Linewidths (Δ*B*_pp_) were in the range of 0.63–0.73 mT (Table 2). The high values of Δ*B*_pp_ indicate that strong dipolar interactions of free radicals exist in melanocytes. Such types of magnetic interactions are characteristic for short-distanced unpaired electrons of free radicals (Wertz and Bolton 1986; Eaton et al. 1998). Chlorpromazine increased the linewidth of EPR spectra of melanocytes in a concentration-dependent manner. It can be concluded that the dipolar interactions rise after chlorpromazine treatment of HEMn-DP cells. Melanin free-radical concentrations in the samples, which are proportional to the integral intensities (*I*) of EPR lines of melanocytes, were higher for cells treated with chlorpromazine in concentrations of 1 × 10^−7^ and 1 × 10^−6^ M than for the control cells (Table 2). This effect was not observed for chlorpromazine concentration 1 × 10^−8^ M, when similar values of integral intensity as for control cells was obtained.

**Fig. 2:**
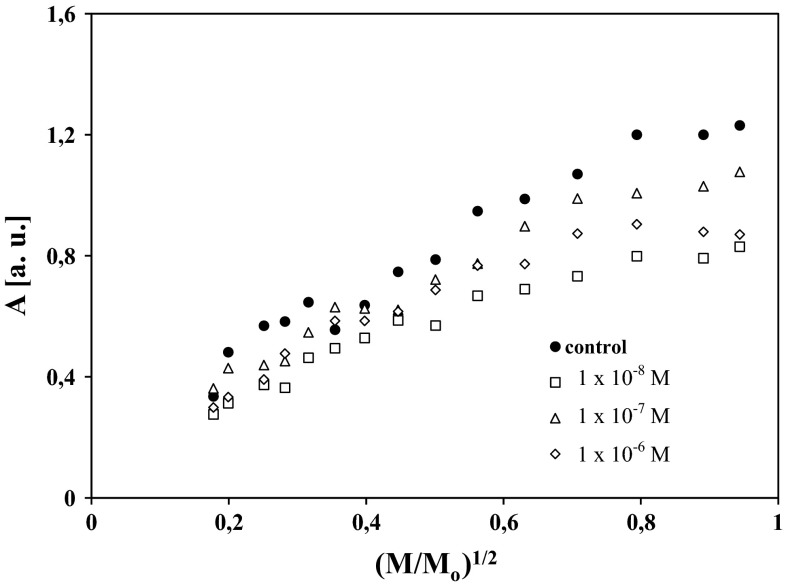
Influence of microwave power (*M*/*M*_0_) on amplitude (*A*) of EPR spectra of HEMn-DP control cells (*filled circle*) and the cells treated with chlorpromazine in concentrations of 1 × 10^−8^ M (*square*), 1 × 10^−7^ M (*triangle*), and 1 × 10^−6^ M (*diamond*). *M*, *M*_0_ are the microwave power used during the measurement of the spectrum and the total microwave power produced by klystron (70 mW), respectively

**Fig. 3:**
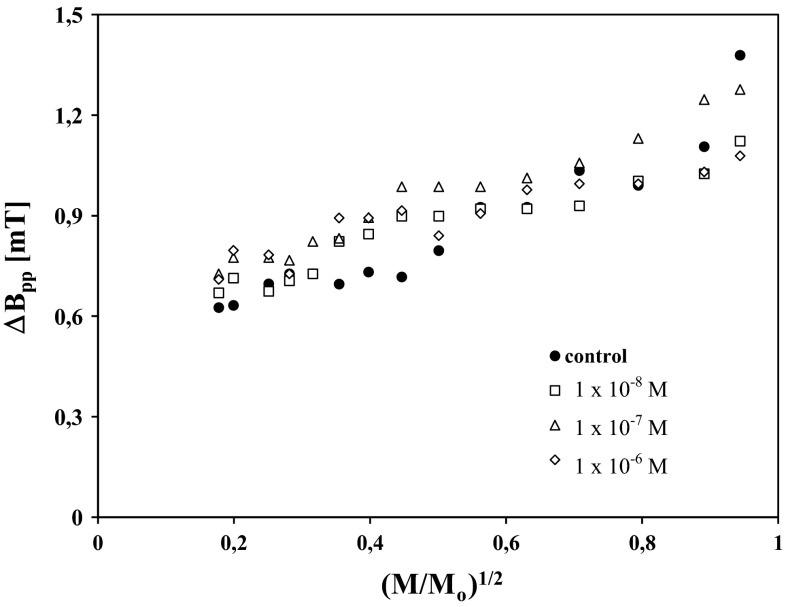
Influence of microwave power (*M*/*M*_0_) on linewidth (Δ*B*_pp_) of EPR spectra of HEMn-DP control cells (*filled circle*) and the cells treated with chlorpromazine in concentrations of 1 × 10^−8^ M (*square*), 1 × 10^−7^ M (*triangle*), and 1 × 10^−6^ M (*diamond*). *M*, *M*_0_ are the microwave power used during the measurement of the spectrum and the total microwave power produced by klystron (70 mW), respectively

Influence of microwave power (*M*/*M*_o_) on amplitude (*A*) and linewidth (Δ*B*_pp_) of EPR spectra of control cells and the cells treated with chlorpromazine at concentrations of 1 × 10^−8^, 1 × 10^−7^, and 1 × 10^−6^ M is presented in Figs. 2 and 3, respectively. The correlations between amplitudes, linewidths, and microwave powers are characteristic for homogeneously broadened EPR lines (Wertz and Bolton 1986; Eaton et al. 1998). The increase of linewidths (Δ*B*_pp_) with increasing microwave power presented in Fig. 3 indicates homogeneous broadening of EPR lines of the tested cells, because for inhomogeneously broadened EPR signals, linewidths do not change with microwave power. The obtained correlations (Figs. 2 and 3) are expected for free radicals homogeneously distributed in the cell samples. Amplitudes (*A*) of the EPR lines of HEMn-DP cells reached maximum at low values of microwave power (<70 mW) (Fig. 2), so slow spin–lattice relaxation processes existed in the tested samples. The slow, but relatively faster spin–lattice relaxation processes existed in control cells and cells treated with chlorpromazine at a concentration of 1 × 10^−8^ M (Fig. 2). Chlorpromazine treatment in concentrations of 1 × 10^−7^ and 1 × 10^−6^ M led to the rise of free radical content in melanocytes (Table 2) and to slower spin–lattice relaxation interactions (Fig. 2). Thus, it may be assumed that the presented increase in free radical concentrations in melanocytes after chlorpromazine treatment plays a critical role in drug toxicity directed to pigmented tissues, as a result of the generation of cellular oxidative stress.

We previously demonstrated that chlorpromazine in concentrations of 1 × 10^−7^ and 1 × 10^−6^ M caused significant alterations in the activity of antioxidant enzymes—superoxide dismutase (SOD), catalase (CAT), and glutathione peroxidase (GPx) in normal human melanocytes (Otręba et al. 2015). Moreover, the analyzed drug increased the cellular H_2_O_2_ level. The results of our prior study (Otręba et al. 2015) and those obtained in this study may explain the role of chlorpromazine in the depletion of antioxidant status in melanocytes, leading to undesirable side effects in vivo.

The performed examination of free radical formation in cells exposed to different chlorpromazine concentrations confirmed the usefulness of electron paramagnetic resonance spectroscopy to determine the effect of a drug on free radical production in a cellular model system in vitro. Moreover, the results that we have obtained show that HEMn-DP cells represent a suitable cell model for a better understanding of the role of melanin biopolymer in undesirable toxic effects directed to pigmented tissues.

## Conclusions

An X-band (9.3 GHz) electron paramagnetic resonance study confirmed the existence of free radicals in normal human melanocytes. It was pointed out that treatment of cells with chlorpromazine in concentrations of 1 × 10^−7^ and 1 × 10^−6^ M caused an increase in the formation of free radicals. This effect was not observed for the lowest drug concentration (1 × 10^−8^ M). Chlorpromazine in higher concentrations generates oxidative stress in melanocytes, which may explain the ability of this drug to induce undesirable toxic effects directed to pigmented tissues.
